# Exercise-induced metabolites: messengers linking epigenetic enzymes and cell remodeling in skeletal muscle regeneration

**DOI:** 10.3389/fphys.2026.1885709

**Published:** 2026-10-30

**Authors:** Wei Wang, Zhao Tian, Churan Bai, Peijin Li, Changjun Zuo, Jin Ye, Zhaowen Li

**Affiliations:** 1 Department of Sports Medicine, The Affiliated Hospital of Wuhan Sports University, Wuhan, China; 2 Wuhan Sports University, Wuhan, China

**Keywords:** epigenetic enzymes, exercise intervention, metabolites, muscle satellite cells, sarcopenia, skeletal muscle regeneration

## Abstract

Skeletal muscle regeneration is a highly coordinated process that depends on muscle satellite cells (MuSCs) and is intricately regulated by metabolic and epigenetic mechanisms. Exercise, recognized as a vital non-pharmaceutical intervention, can significantly enhance the regenerative capacity of skeletal muscle; however, the underlying molecular mechanisms remain inadequately understood. Recent studies suggest that the metabolic products generated during exercise are not merely by-products of energy metabolism; rather, they act as essential regulatory factors involved in epigenetic reprogramming. These metabolites, which include lactate, α-ketoglutarate (α-KG), acetyl-CoA, nicotinamide adenine dinucleotide (NAD^+^), and S-adenosylmethionine (SAM), serve as substrates, cofactors, or signaling molecules. They directly influence the activities of epigenetic enzymes such as histone acetyltransferases, deacetylases, demethylases, and DNA methyltransferases. This mechanism converts dynamic changes in metabolic states into stable chromatin modifications and gene expression programs, thereby affecting the activation, proliferation, and differentiation of MuSCs. This article presents a systematic review of the regulatory mechanisms that govern the metabolic-epigenetic axis during skeletal muscle regeneration. It emphasizes the role of exercise-induced metabolites in facilitating cell reprogramming through chromatin remodeling. Additionally, the article proposes an integrated theoretical framework that underscores the dynamic coordination of metabolic signals and their time-dependent characteristics throughout various stages of regeneration. Furthermore, it explores potential clinical translational strategies aimed at targeting metabolic-epigenetic coupling, including personalized exercise interventions and metabolic supplementation regimens. A comprehensive understanding of this regulatory network is expected to provide a new theoretical foundation for the precise treatment of muscle injuries, age-related sarcopenia, and metabolic diseases.

## Introduction

Skeletal muscle, the largest motor and metabolic organ in the human body, exhibits remarkable regenerative capacity, which is vital for sustaining motor function and metabolic homeostasis (Yin et al., 2013; Chung et al., 2025). This regeneration process is heavily dependent on the precise regulation of muscle stem cells (MuSCs). Under physiological conditions, MuSCs undergo a complex series of transformations, including activation, proliferation, differentiation from the resting state, and eventual fusion into mature muscle fibers. This process is regulated by the canonical myogenic regulatory cascade, characterized by the sequential expression and functional coupling of Pax7, MyoD, and myogenin (Rahman et al., 2023; Tsuji et al., 2025) However, factors such as aging, trauma, and various chronic diseases can impair MuSC function, leading to diminished regenerative efficiency and resulting in muscle atrophy, fibrosis, and motor dysfunction (Sousa-Victor et al., 2014; Sartorelli and Ciuffoli, 2025). Therefore, a comprehensive analysis of the molecular mechanisms that govern MuSC fate is essential for developing effective clinical intervention strategies.

In this review, “exercise” is defined as structured physical activity and is primarily categorized into two major modalities: endurance (aerobic) exercise and resistance (strength/load-bearing) exercise. Endurance exercise typically involves prolonged, low-to-moderate intensity activity that predominantly relies on oxidative metabolism, whereas resistance exercise is characterized by high-intensity mechanical loading that promotes anabolic signaling and muscle hypertrophy (Hughes et al., 2018; Zhao and Gao, 2024).Importantly, these modalities generate distinct metabolic environments and differentially regulate MuSC behavior. Endurance exercise primarily influences MuSC differentiation and metabolic adaptation, while resistance exercise promotes MuSC activation, proliferation, and myonuclear accretion, which are essential for muscle hypertrophy. Recent studies, including work by Kevin Murach and colleagues, have highlighted load-induced transcriptional and epigenetic programs in MuSCs, emphasizing the importance of mechanical stimuli in shaping regenerative outcomes (Fukada and Nakamura, 2021; Murach et al., 2021).These modality-specific differences provide a critical framework for understanding how exercise-induced metabolites interact with epigenetic mechanisms to regulate skeletal muscle regeneration.

Exercise is currently recognized as the most effective non-pharmaceutical strategy for promoting skeletal muscle regeneration and mitigating muscle aging (Egan and Sharples, 2023). Traditional views primarily attribute its effects to mechanical load stimulation, growth factor release, and improvements in the immune microenvironment (Hawley et al., 2014). However, advancements in metabolomics and epigenomics have led the academic community to increasingly acknowledge that exercise-induced metabolic reprogramming plays a crucial role in regulating the fate of muscle stem cells (MuSCs) (Zheng et al., 2025). Exercise not only alters energy supply patterns but also acts as a “messenger” for information transmission both within and outside cells by generating a variety of bioactive metabolites (Chow et al., 2022; Gad et al., 2024; Walzik et al., 2024). Recent studies suggest that metabolites are no longer regarded merely as energy substrates or by-products; rather, they are recognized as essential regulators of epigenetic enzyme activity (Zhang et al., 2019; Desgeorges et al., 2024). Key metabolites such as lactate, acetyl-coenzyme A, α-KG, NAD^+^, and SAM function as substrates, cofactors, or competitive inhibitors that directly influence the activities of histone acetyltransferases, deacetylases, demethylases, and DNA methyltransferases (Dai et al., 2023; Meng et al., 2025). This “metabolic-epigenetic axis” translates systemic metabolic fluctuations induced by exercise into stable chromatin remodeling and gene transcription programs within the cell nucleus, thereby precisely orchestrating the activation and functional execution of MuSCs (Yucel et al., 2019).

Most existing studies concentrate on the effects of individual metabolites or enzymes, often lacking a temporal dynamic integration framework that considers multi-metabolite competition and synergy during exercise (Xu et al., 2023). This paper systematically reviews recent advancements in exercise-induced metabolites associated with skeletal muscle regeneration. It introduces, for the first time, a time-dependent metabolic-epigenetic integration framework and discusses the variations in metabolite profiles across different stages of regeneration, as well as their interactions with the AMPK/ROS/CaMK signaling pathways. Furthermore, the paper explores the clinical translational potential of this network in the context of sarcopenia and muscle injury repair, thereby providing theoretical support for precise exercise prescriptions and metabolic intervention strategies. To provide an overview of the metabolic–epigenetic axis in skeletal muscle regeneration, the core framework is summarized in **Figure 1**.

**Figure 1:**
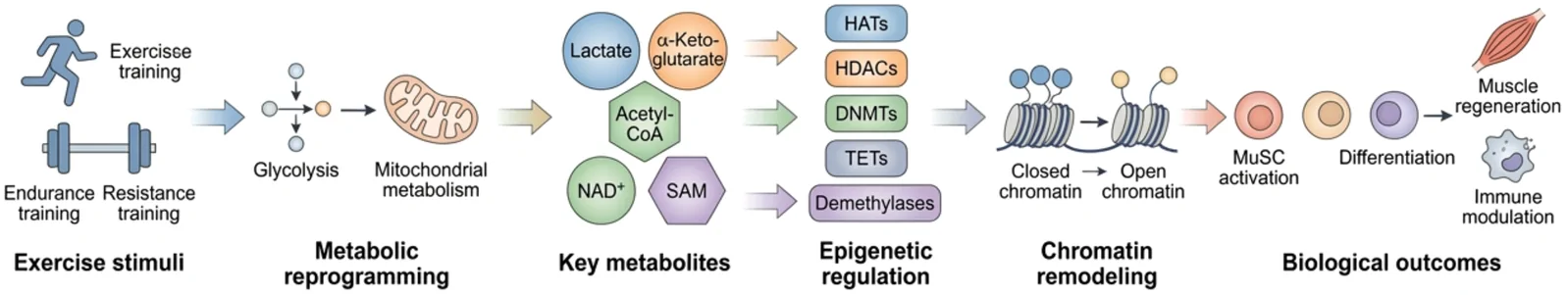
Exercise-induced metabolic–epigenetic coupling governs skeletal muscle regeneration. Graphical overview of the metabolism–epigenetic axis in skeletal muscle regeneration. Exercise induces metabolic reprogramming, generating key metabolites—including lactate, α-ketoglutarate (α-KG), acetyl-CoA, NAD^+^, and S-adenosylmethionine (SAM). These metabolites function as substrates or signaling molecules to regulate epigenetic enzymes and chromatin accessibility, thereby governing MuSC activation, proliferation, and differentiation, while modulating the local immune microenvironment to promote muscle regeneration.

## The metabolic-epigenetic axis in skeletal muscle regeneration

Skeletal muscle regeneration is a highly coordinated process driven by MuSCs, which undergo a well-defined progression from quiescence to activation, proliferation, and differentiation. Quiescent MuSCs are characterized by the expression of Pax7. Upon activation, MuSCs co-express Pax7 and MyoD, entering a proliferative myoblast state (Seaborne et al., 2018; Williams et al., 2021). As differentiation proceeds, Pax7 expression declines, while MyoD and myogenin become dominant, marking terminal differentiation into myocytes. These myocytes subsequently fuse to form new myofibers or repair damaged tissue.

Importantly, beyond contributing to newly formed fibers, a subset of activated MuSCs provides additional myonuclei to existing muscle fibers. Emerging evidence indicates that these myonuclei are not homogeneous but exhibit nucleus type–specific epigenetic signatures, reflecting their cellular origin and prior adaptive history. Recent studies have demonstrated that DNA methylation patterns differ between myonuclei and other nuclear populations within skeletal muscle, revealing an epigenetic “memory” of prior adaptations (Wen et al., 2021). Moreover, lifelong physical activity induces a MuSC-dependent epigenetic fingerprint in muscle identity genes, suggesting that MuSCs play a central role in establishing and maintaining long-term transcriptional programs associated with muscle function and plasticity (Murach et al., 2025). These findings highlight that epigenetic memory is both nucleus-specific and stem cell–dependent, providing a mechanistic basis for sustained adaptive responses such as hypertrophy and regeneration.

During this regenerative process, MuSCs undergo dynamic metabolic reprogramming that is tightly coupled to their fate transitions. Oxidative phosphorylation predominates in the quiescent state, whereas glycolysis is markedly upregulated during the activation and proliferation phases to support rapid energy demand and biosynthetic processes. This metabolic shift resembles a “Warburg-like” effect, in which cells preferentially rely on glycolysis even in the presence of oxygen to sustain growth-related processes. Importantly, such metabolic reprogramming reflects a fundamental principle of cell growth rather than a pathological feature, as highlighted by the concept that “cell growth is cell growth” (Burke et al., 2024). In skeletal muscle, this glycolytic reprogramming supports the synthesis of macromolecules and provides intermediates required for epigenetic regulation, thereby facilitating efficient regeneration. Furthermore, emerging evidence suggests that hypertrophying muscle fibers may partially reprogram their metabolism in a manner analogous to cancer cells; however, this process is tightly controlled and functionally beneficial, contributing to muscle repair and adaptation rather than uncontrolled proliferation (Wackerhage et al., 2022). Following this proliferative phase, mitochondrial oxidative metabolism is progressively restored during differentiation, reflecting a transition toward a more energy-efficient and functionally mature state (Lu et al., 2026). This stage-specific metabolic remodeling not only satisfies energetic requirements but also plays a pivotal role in regulating epigenetic modifications through the availability of key metabolic intermediates (Jacques et al., 2019).

Specifically, increased glycolytic flux leads to lactate accumulation, which promotes histone lactylation and facilitates gene expression associated with regeneration. Meanwhile, α-ketoglutarate (α-KG), generated from the tricarboxylic acid cycle, serves as an essential cofactor for dioxygenases involved in DNA and histone demethylation. Acetyl-CoA acts as a substrate for histone acetylation, thereby enhancing chromatin accessibility and transcriptional activation. In contrast, NAD^+^ regulates Sirtuin-mediated deacetylation, linking cellular redox status to transcriptional control. DNA and histone methylation are key epigenetic mechanisms that regulate gene expression by modulating chromatin accessibility. DNA methylation primarily occurs at CpG dinucleotides, particularly within promoter regions, where hypermethylation is generally associated with transcriptional repression, whereas hypomethylation is linked to gene activation. In contrast, histone methylation exhibits context-dependent regulatory roles: for example, H3K4me3 is associated with transcriptional activation, whereas H3K27me3 and H3K9me3 are typically linked to gene silencing (Black et al., 2012; Jones, 2012; Hyun et al., 2017).

During skeletal muscle regeneration, methylation changes are highly dynamic and locus-specific rather than uniformly distributed across the genome. Early activation stages of MuSCs are often associated with demethylation of myogenic regulatory genes, whereas later stages involve selective remethylation to stabilize transcriptional programs and support terminal differentiation (Dilworth and Blais, 2011; Segalés et al., 2015).

Additionally, S-adenosylmethionine (SAM) functions as the primary methyl donor for DNA and histone methylation, thereby influencing gene silencing and lineage commitment (Verma and Lindroth, 2025; Wei et al., 2025).

Through these coordinated processes, metabolic reprogramming directly shapes the epigenetic landscape, converting transient metabolic cues into stable gene expression programs that ultimately determine MuSC fate decisions (Sun et al., 2025). Notably, these regulatory mechanisms are highly stage-specific, locus-dependent, and nucleus type–specific, reflecting the precise temporal and spatial control of gene expression required for effective muscle regeneration.

Collectively, this tightly integrated mechanism—referred to as the “metabolic–epigenetic axis”—represents a fundamental molecular basis of skeletal muscle regeneration. Importantly, this framework also provides a mechanistic explanation for how exercise-induced metabolic signals may establish long-lasting epigenetic memory and thereby influence future adaptive responses. This mechanism of “metabolic-epigenetic coupling” constitutes a critical molecular basis for skeletal muscle regeneration. This metabolism–epigenetic coupling mechanism is summarized in **Figure 2**. A summary of key metabolic–epigenetic interactions is provided in **Table 1**.

**Figure 2:**
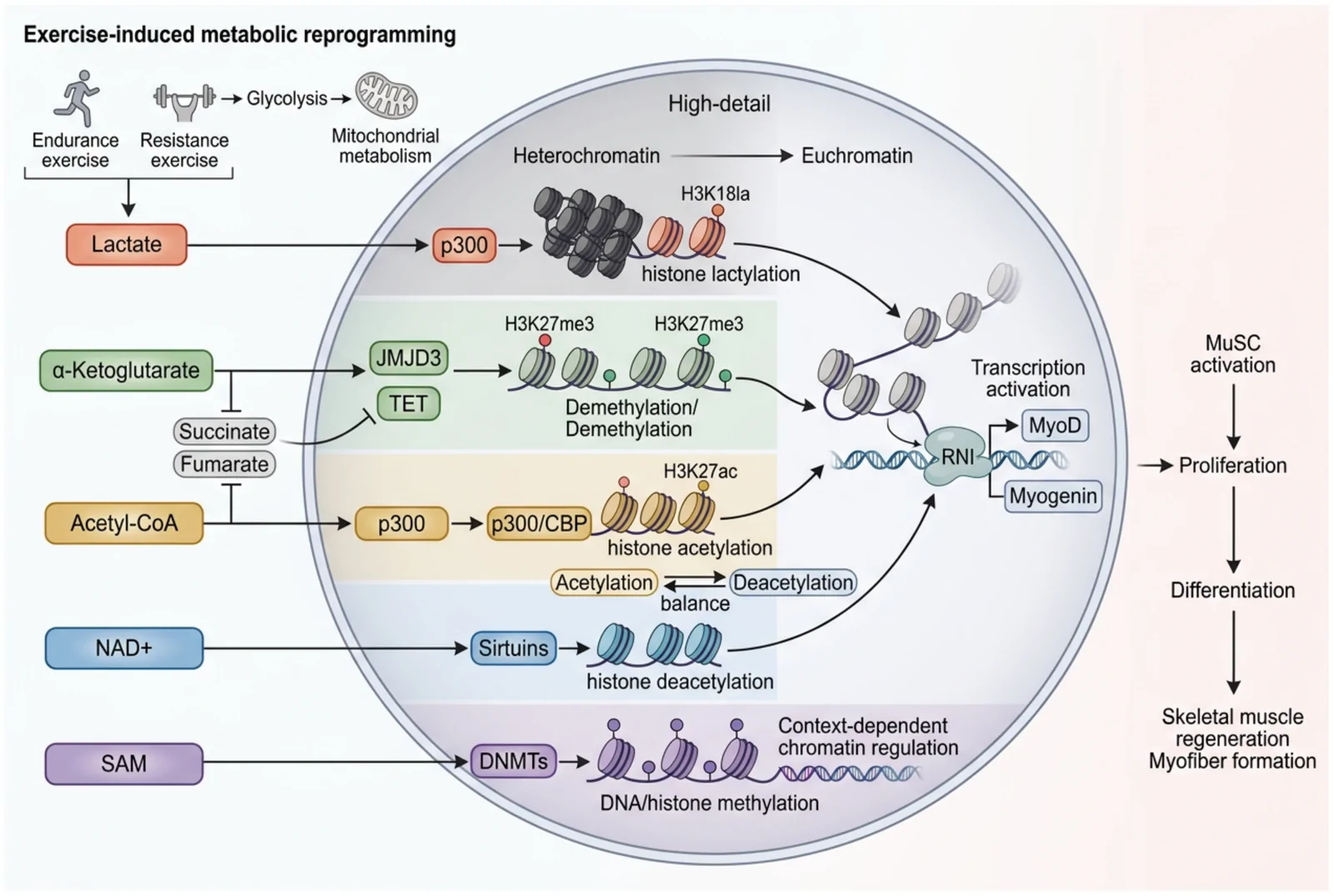
Metabolite-driven epigenetic mechanisms regulating muscle stem cell fate. Mechanistic framework of metabolite-mediated epigenetic regulation during skeletal muscle regeneration. Exercise-induced metabolites function as essential substrates or cofactors for diverse epigenetic enzymes. Specifically, lactate promotes histone lactylation, α-KG facilitates demethylation, acetyl-CoA supports histone acetylation, NAD^+^ regulates Sirtuin-mediated deacetylation, and SAM provides methyl groups for DNA and histone methylation. These coordinated metabolic-epigenetic interactions dynamically regulate chromatin accessibility and downstream gene expression, thereby determining MuSC fate transitions.

**Table 1:** Metabolic–epigenetic regulation of MuSC fate during skeletal muscle regeneration.

Metabolite	Metabolic source	Epigenetic mechanism	MuSC functional outcome	Exercise modality	References
Lactate	Glycolysis	Histone lactylation	Activation and early regeneration	Endurance exercise	(Zhang et al., 2019)
α-KG	TCA cycle	DNA and histone demethylation	Differentiation	Endurance exercise	(Murach et al., 2025)
Acetyl-CoA	Glycolysis/TCA cycle	Histone acetylation	Proliferation and hypertrophy	Resistance exercise	(Murach et al., 2021)
NAD^+^	Cellular redox metabolism	Sirtuin-mediated deacetylation	Quiescence maintenance and metabolic adaptation	Endurance exercise	(Murach et al., 2021)
SAM	Methionine cycle	DNA and histone methylation	Lineage commitment and gene silencing	Both modalities	(Murach et al., 2021; Murach et al., 2025)

Metabolites generated during exercise-induced metabolic reprogramming regulate epigenetic modifications and influence MuSC fate.

## Exercise metabolites: epigenetic messengers of skeletal muscle regeneration

Exercise-induced metabolic reprogramming not only modifies cellular energy metabolism but also converts metabolic states into molecular signals that the epigenetic system can recognize by regulating the production and distribution of key metabolic intermediates (Jacques et al., 2019). Recent studies demonstrate that lactate, α-KG, acetyl-coenzyme A, NAD^+^, and SAM function not only as metabolic intermediates but also as direct regulators of histone modification and DNA methylation processes. They accomplish this by acting as substrates, cofactors, or heterosteric regulatory molecules, thereby reprogramming gene expression at the chromatin level (Verma and Lindroth, 2025). Importantly, these metabolites do not function in isolation. Instead, through the combined effects of spatiotemporal dynamics, concentration gradients, and metabolic competition, they create a highly integrated “metabolic-epigenetic regulatory network” that precisely governs the fate and regeneration of MuSC (Sartorelli and Ciuffoli, 2025; Wei et al., 2025).

### Lactate: mediating histone lactylation and regulating immune homeostasis

Historically, lactate has been recognized as a key metabolic intermediate in the Cori cycle, where lactate produced in skeletal muscle during exercise is transported to the liver and converted back into glucose via gluconeogenesis. This inter-organ metabolic shuttle, first described by Cori and Cori, highlights the fundamental role of lactate in maintaining systemic energy homeostasis (Gladden, 2004; Brooks, 2018). Although lactate was traditionally viewed as a metabolic byproduct, it is now increasingly recognized as an important regulator of cellular signaling and gene expression. This classical understanding provides a conceptual foundation for the emerging role of lactate in epigenetic regulation. Recent studies have demonstrated that lactate can directly influence chromatin dynamics through histone lactylation, thereby linking metabolic flux to transcriptional control and cellular reprogramming (Zhang et al., 2019). In this context, lactate should not only be considered a metabolic intermediate but also a key mediator connecting exercise-induced metabolic changes with epigenetic regulation during skeletal muscle regeneration.

During exercise, enhanced glycolysis leads to the rapid accumulation of lactate, which functions not only as a by-product of energy metabolism but also as a crucial signaling molecule involved in epigenetic regulation. Mechanistically, lactate mediates histone lactylation via acyltransferases such as p300, as exemplified by H3K18la, thereby facilitating chromatin remodeling and enhancing gene transcriptional activity (Meng et al., 2025; Sun et al., 2025). In addition to its direct modification of histones, lactate can indirectly affect Sirtuin activity by modulating the NAD^+^/NADH ratio, establishing cross-regulation with the deacetylation pathway (Nalbandian et al., 2020; Walzik et al., 2023). At the level of target genes, lactate modification is enriched in the promoter regions of genes such as Neu2 and Arg1, promoting their expression (Dai et al., 2023; Desgeorges et al., 2024). Furthermore, lactate plays a dual role in the regeneration of skeletal muscle; it facilitates the differentiation of MuSCs and the initiation of myogenic programs (Meng et al., 2025). Second, it promotes the polarization of macrophages toward the M2 phenotype, which inhibits inflammatory responses and enhances the regenerative microenvironment (Zhang et al., 2020). The effects of lactate exhibit a distinct dependence on both dose and duration; moderate increases facilitate regeneration, while excessive accumulation can lead to acidosis and hinder regeneration (Wang and Zhu, 2025). Moreover, lactate may broaden its epigenetic regulatory influence by affecting DNA methylation and non-coding RNA networks (Zhou et al., 2025). Through modifications and metabolism-redox interactions, lactate converts glycolytic enhancement signals into multi-level epigenetic regulatory signals, thereby fulfilling a dual role in promoting differentiation and regulating immune responses during muscle regeneration.

### α-KG: driving demethylation and chromatin opening

Exercise enhances mitochondrial oxidative metabolism and stimulates the activation of the tricarboxylic acid cycle, leading to increased production of α-KG (Weidenhamer et al., 2025). As a vital cofactor for histone demethylases in the JmjC domain and DNA demethylases in the TET family, α-KG significantly contributes to epigenetic regulation (Naeini et al., 2023). Mechanistically, α-KG enhances chromatin accessibility and activates myogenic genes, such as MyoD and Myogenin, by facilitating the removal of inhibitory methylation marks, including H3K27me3 and H3K9me3 (Ciuffoli et al., 2024). Importantly, a competitive inhibitory relationship exists between α-KG and the metabolites succinate and fumarate. These metabolites act as “inhibitory metabolic signals,” hindering α-KG-dependent demethylation reactions and thereby establishing a metabolic regulatory balance (Sahu and Lu, 2025). Furthermore, α-KG is believed to play a crucial role in regulating the redox state and reactive oxygen species (ROS) levels within cells, which indirectly influences the activity of epigenetic enzymes (Milanović et al., 2024). Functionally, α-ketoglutarate (α-KG) facilitates the transition of muscle stem cells (MuSCs) from a proliferative state to differentiation by promoting demethylation reactions that sustain their epigenetic plasticity (Ciuffoli et al., 2024). α-KG not only drives these demethylation reactions but also creates a competitive axis with inhibitory metabolites, thereby transforming mitochondrial metabolic states into signals that activate chromatin. This mechanism positions α-KG as a crucial link between energy metabolism and gene activation.

### Acetyl-CoA: regulation of histone acetylation and transcriptional activation

During exercise, the metabolism of sugars and the oxidation of fatty acids are enhanced, resulting in increased production of acetyl-CoA (Miranda-Cervantes et al., 2025). As a direct substrate for histone acetylation reactions, the concentration of acetyl-CoA significantly influences the extent of chromatin acetylation (Yu et al., 2024). Mechanistically, acetyl-CoA facilitates the formation of active markers, such as H3K27ac and H3K9ac, by supplying acetyl groups to histone acetyltransferases, including p300/CBP (Takatoya et al., 2025; Xu L. et al., 2025). Recent studies have demonstrated that acetyl-CoA is produced not only in the cytoplasm and mitochondria but also locally within the nucleus through pathways such as ATP-citrate lyase (ACLY), thus enabling precise regulation of specific gene loci (Lazaropoulos et al., 2024). A dynamic balance exists between acetyl-CoA and the NAD^+^-Sirtuin pathway; the competition between acetylation and deacetylation determines the chromatin state, thereby establishing a refined regulatory network (Xu M. et al., 2025). Functionally, acetyl-CoA enhances the proliferation and differentiation of MuSC by upregulating the expression of genes such as MyoD and PGC-1α, while also promoting mitochondrial biogenesis and metabolic adaptation (Sartorelli and Ciuffoli, 2025). Acetyl-CoA regulates nuclear acetylation levels and maintains a dynamic equilibrium with the deacetylation pathway, transforming metabolic underflow dynamics into precise transcriptional activation signals.

### NAD^+^: integration of energy metabolism and deacetylation through the Sirtuins family

During exercise, the energy demands of cells increase, resulting in dynamic changes in the NAD^+^/NADH ratio. This alteration positions NAD^+^ as a critical link between metabolic status and epigenetic regulation. Mechanistically, NAD^+^ functions as an essential cofactor for the Sirtuin family of deacetylases, which influence gene expression and cellular metabolic adaptation by modulating the acetylation status of both histone and non-histone proteins, including PGC-1α and FOXO (Yusri et al., 2025). Additionally, fluctuations in NAD^+^ levels can participate in DNA repair and chromatin structure regulation by affecting PARP activity (Ortega et al., 2025). Furthermore, NAD^+^ metabolism is intricately connected to lactate production and redox status, establishing a cross-regulatory network with glycolysis and mitochondrial metabolism. Functionally, NAD^+^ enhances the regenerative environment of MuSCs and maintains cellular homeostasis by promoting mitochondrial function, antioxidant responses, and the regulation of inflammation (Su et al., 2024). NAD^+^ is vital for muscle regeneration, as it modulates pathways such as Sirtuin and PARP, thereby integrating cellular energy and redox states into epigenetic regulatory signals.

### SAM: maintaining methylation homeostasis and epigenetic memory formation

Exercise modulates the production of S-adenosylmethionine (SAM) by influencing the one-carbon metabolic pathway, which subsequently regulates cellular methylation capacity. As the primary methyl donor in DNA and histone methylation, SAM is essential for maintaining epigenetic homeostasis. Mechanistically, SAM enables gene expression silencing or activation by providing methyl groups to DNA methyltransferases (DNMT) and histone methyltransferases (Xing and Tu, 2025). Moreover, the ratio of SAM to S-adenosylhomocysteine (SAH) is crucial for determining the efficiency of methyl transfer reactions and serves as a significant indicator of cellular methylation potential. Additionally, SAM-mediated methylation modifications exhibit relative stability and can be propagated during cell division, thereby establishing “epigenetic memory.” This mechanism may clarify the prolonged effects of exercise on the long-term adaptation and regenerative capacity of skeletal muscle (Pilotto et al., 2025). Functionally, SAM stabilizes the gene expression program associated with muscle stem cell (MuSC) differentiation and contributes to the long-term adaptation process (Kang et al., 2024). SAM transforms the one-carbon metabolic state into a stable gene expression pattern by regulating methylation reactions and facilitating the formation of epigenetic memory.

In summary, exercise-induced metabolites have established a dynamic metabolic-epigenetic regulatory network by serving as substrates, cofactors, and regulatory molecules. Lactate plays a predominant role in early stress responses and immune regulation, while acetyl-coenzyme A and NAD^+^ are essential for mid-term transcriptional activation and metabolic adaptation. In the later stages, α-KG and SAM contribute to gene activation and the maintenance of epigenetic stability. This “hierarchical regulatory model” elucidates the sequential roles of metabolic signals throughout various stages of regeneration, providing a systematic framework for understanding how exercise promotes skeletal muscle regeneration.

## Temporal evolution and signal integration of the metabolic-epigenetic axis

The aforementioned research indicates that various metabolites induced by exercise contribute to skeletal muscle regeneration by regulating epigenetic modifications. However, these metabolic signals do not operate in isolation; instead, they form a highly integrated regulatory system that functions at both spatiotemporal and metabolic network levels. Current studies increasingly recognize that skeletal muscle regeneration is fundamentally a dynamic regulatory process driven by metabolism and mediated by epigenetics. A defining characteristic of this process is the synergy, competition, and phase-specific dominance among various metabolites (Lu et al., 2026). The temporal dynamics of metabolite-driven epigenetic regulation are illustrated in **Figure 3**.

**Figure 3:**
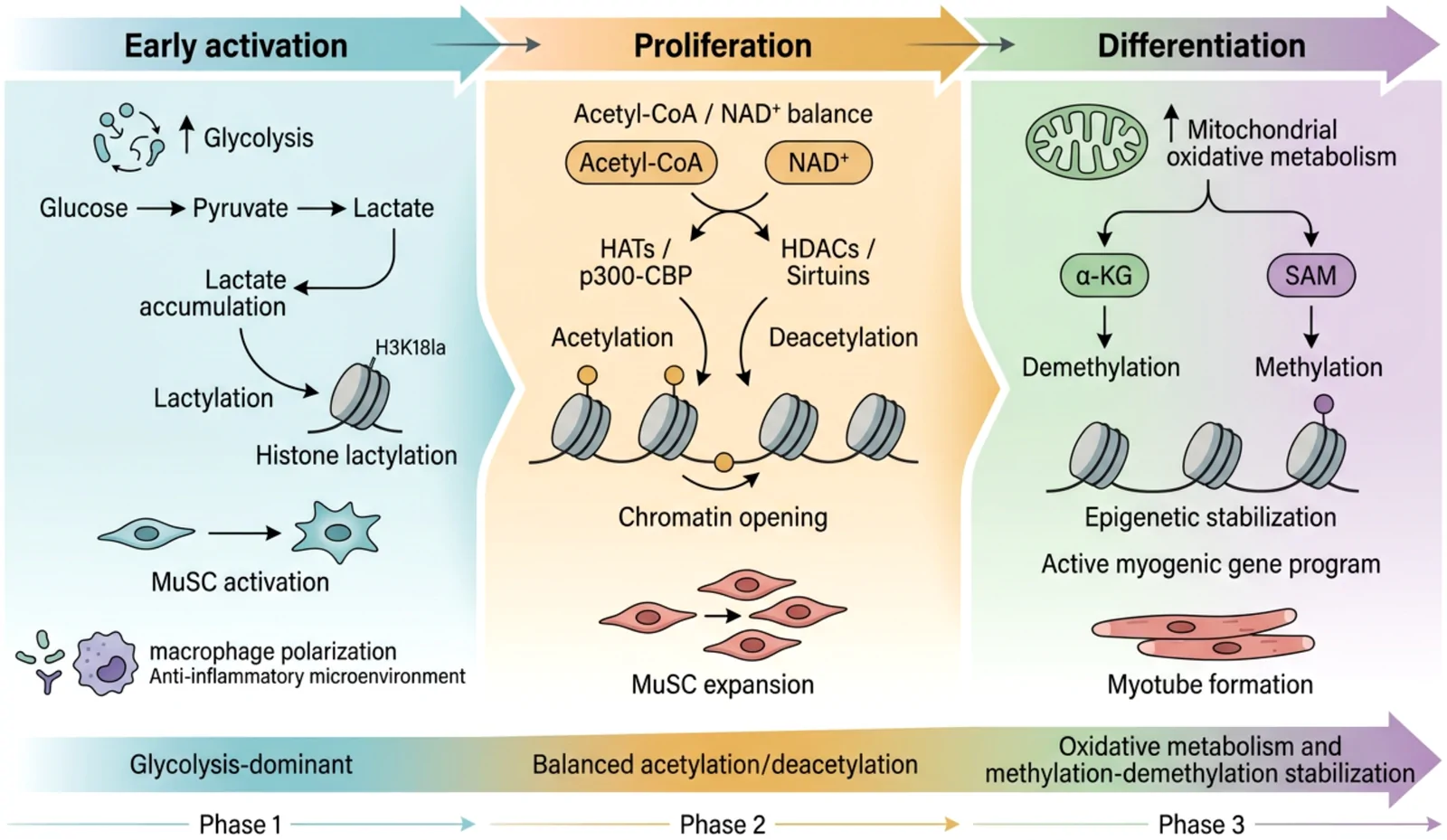
Stage-specific metabolic–epigenetic remodeling during skeletal muscle regeneration.Temporal dynamics of metabolic and epigenetic regulation throughout the myogenic repair process. Distinct metabolic and epigenetic signatures characterize the progression of muscle regeneration. The early activation phase is primarily driven by lactate-mediated histone lactylation; the proliferation phase involves coordinated regulation of chromatin accessibility by acetyl-CoA and NAD^+^; whereas the differentiation phase is characterized by α-KG and SAM-mediated methylation/demethylation, which stabilize gene expression programs to facilitate myofiber maturation.

### Phasic metabolic characteristics and epigenetic regulation of skeletal muscle regeneration

During the early activation stage, an explosion of glycolytic flux results in the rapid accumulation of lactate. Lactate facilitates the transition of MuSCs from a resting state to an activated state by mediating histone lactylation, such as H3K18la, and by regulating the immune microenvironment. In the metaphase proliferation stage, a dynamic equilibrium system composed of acetyl-CoA and NAD^+^ governs chromatin accessibility, thereby supporting the rapid expansion of MuSC. In the late differentiation stage, mitochondrial oxidative metabolism prevails, accompanied by increased levels of α-KGand SAM. By precisely erasing inhibitory markers and maintaining methylation homeostasis, these metabolites collectively ensure epigenetic fidelity and optimize the regulatory threshold for gene activation, thereby promoting the transformation of MuSC into mature muscle fibers.

### Differential shaping of the apparent remodeling trajectory by motion patterns

Variations in metabolite profiles arising from distinct exercise regimens shape the specific trajectory of epigenetic remodeling. Endurance training significantly activates the AMPK-ROS signaling pathway, characterized by an increased NAD+/NADH ratio and α-KG accumulation (Reisman et al., 2024). At the epigenetic level, this activation enhances SIRT1-mediated deacetylation and JMJD3-mediated H3K27me3 demethylation (Li et al., 2024). This pattern is particularly critical during the later stages of MuSC differentiation, as it can substantially upregulate metabolic genes such as PGC-1α, thereby promoting mitochondrial biogenesis and facilitating the phenotypic transformation of slow muscle fibers. In contrast, resistance training primarily activates the mTORC1-CaMKII signaling network, directing metabolic flow toward the high-throughput supply of acetyl-CoA and SAM (Mănescu, 2026). This activation leads to elevated histone acetylation, including H3K27ac, and the maintenance of DNA methylation homeostasis at the epigenetic level (Lim et al., 2020; Silva et al., 2025). This model predominantly influences the proliferation and fusion stages of MuSCs, thereby promoting skeletal muscle hypertrophy and hypertrophic remodeling following regeneration by enhancing the transcriptional activity of MyoD and genes associated with protein synthesis.

### Competitive synergy and apparent homeostasis among metabolites

In addition to temporal variations, metabolites collectively influence the status of epigenetic modifications through mechanisms of competition and synergy. Within the regulation of demethylation, a competitive relationship exists among α-KG, succinic acid, and fumaric acid. The latter two compounds inhibit α-KG-dependent demethylase activity, thereby restricting the chromatin deinhibition process and establishing a “braking mechanism” at the metabolic level (Xiao et al., 2012; Chaves-Perez et al., 2025). Regarding acetylation regulation, acetyl-CoA and NAD^+^ facilitate acetylation and deacetylation reactions, respectively. The relative concentrations of these metabolites dictate the open and closed states of chromatin, creating a dynamic equilibrium system (Sahu and Lu, 2025; Verma and Lindroth, 2025). In the context of methylation regulation, the ratio of SAM to its metabolite SAH determines the efficiency of methylation transfer reactions, which subsequently influences the methylation levels of DNA and histones (Xing and Tu, 2025). Importantly, these metabolic competitive relationships do not function in isolation; rather, they form intricate networks through interconnections among various metabolic pathways. For instance, increased glycolysis raises lactate levels and impacts NAD^+^ regeneration, thereby indirectly regulating the deacetylation reaction. Metabolites collaboratively establish a “dynamic balance system” of epigenetic regulation through both competition and synergy, thus precisely modulating the chromatin state.

### Integrated regulation of the metabolic-epigenetic axis by multidimensional signaling pathways

In addition to the direct effects of metabolites, metabolic-epigenetic regulation is influenced by various signaling pathways. Energy-sensing pathways, such as AMPK, are activated during exercise and can alter the levels of acetyl-CoA and NAD^+^ by modulating the activity of metabolic enzymes. Furthermore, these pathways directly affect epigenetic enzymes, thereby regulating chromatin states (Mănescu et al., 2026). Conversely, the mTOR signaling pathway indirectly influences epigenetic modifications through its role in regulating protein synthesis and metabolic reprogramming. Reactive oxygen species (ROS) and calcium ion (Ca²^+^) signaling undergo significant changes during exercise. These factors can indirectly modulate the levels of α-KG and NAD^+^ by affecting mitochondrial function and the activity of metabolic enzymes, thereby influencing demethylation and deacetylation processes (Zhou et al., 2024; Mănescu et al., 2026). Immune signals also contribute; for instance, inflammatory factors can further impact epigenetic modifications and regeneration processes by altering lactate metabolism and the polarization state of macrophages (Li et al., 2026). The relationship between metabolism and epigenetic regulation is not linear; rather, it intersects with energy sensing, redox, and immune signaling to form a multi-level regulatory network.

### Long-term exercise-induced “epigenetic memory” and functional adaptation

Recent studies suggest that exercise-induced metabolic changes may establish a “memory effect” through epigenetic modifications, thereby influencing the long-term regenerative capacity of skeletal muscle (Widmann et al., 2019). Mechanistically, DNA methylation and histone modifications exhibit a degree of stability and can be inherited during cell division, enabling muscle stem cells (MuSCs) to demonstrate enhanced responsiveness to subsequent stimuli. This “epigenetic memory” is likely associated with the improved regenerative capacity observed following exercise training (Pilotto et al., 2025). Furthermore, repeated fluctuations in metabolic status may progressively alter the epigenetic landscape by continuously modulating the levels of S-adenosylmethionine (SAM) and α-ketoglutarate (α-KG), thus facilitating long-term adaptation. The interplay between metabolism and epigenetics not only contributes to immediate regulatory processes but may also mediate long-term regenerative adaptations through memory effects.

A multi-level integrated regulatory system develops between metabolism and epigenetics during the regeneration of skeletal muscle. Various metabolites play a pivotal role at distinct phases, creating a dynamic equilibrium through competition and collaboration across spatial and network dimensions. Additionally, these metabolites interact with multiple signaling pathways, collectively shaping the fate transformation and tissue regeneration of muscle stem cells (MuSCs). This integrated model deepens our understanding of the mechanisms by which exercise promotes skeletal muscle regeneration and provides a theoretical foundation for metabolic interventions and targeted therapies.

## Clinical prospects: from pathological disorders to precise intervention strategies

The exercise-induced metabolic-epigenetic coupling mechanism in skeletal muscle regeneration has garnered attention. However, this regulatory network frequently becomes disrupted under various pathological conditions, negatively impacting muscle repair capabilities. Conditions such as muscle injury, age-related sarcopenia, and metabolic myopathy are often characterized by abnormal metabolic reprogramming and imbalances in epigenetic regulation. Therefore, the development of intervention strategies focused on the “metabolic-epigenetic axis” may provide a novel approach for treating skeletal muscle-related diseases. A schematic overview of clinical intervention strategies targeting this axis is presented in **Figure 4**.

**Figure 4:**
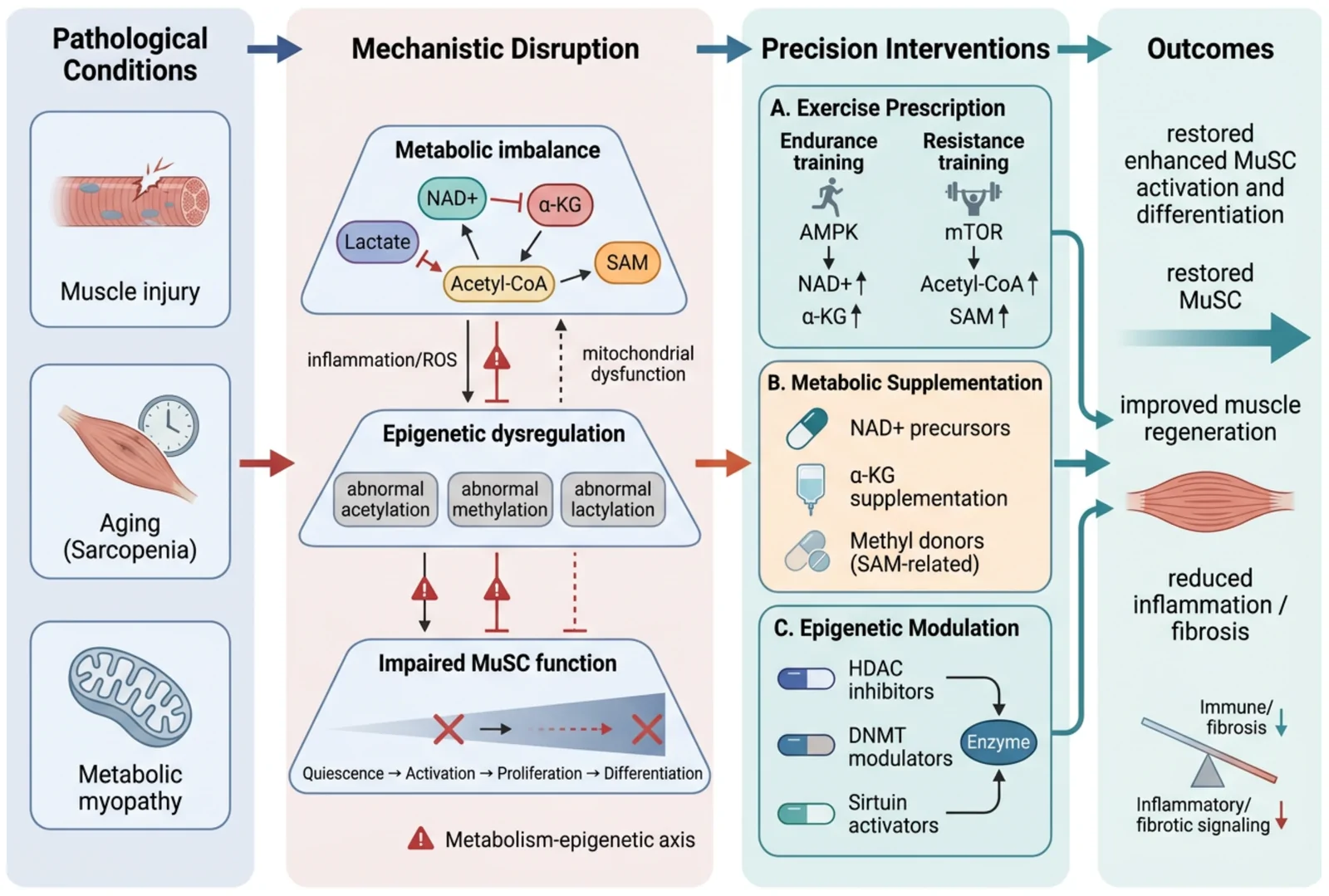
Precision therapeutic strategies targeting the metabolic–epigenetic axis for skeletal muscle regeneration. Clinical translation framework targeting the metabolic–epigenetic axis in skeletal muscle regeneration. Pathological conditions (e.g., muscle injury, sarcopenia, and myopathy) disrupt metabolic and epigenetic homeostasis, leading to impaired MuSC function. Precision interventions—including tailored exercise prescriptions, metabolic supplementation, and targeted epigenetic modulation—aim to restore regulatory balance, thereby enhancing skeletal muscle regeneration and functional recovery.

During muscle injury, inflammatory responses are intricately associated with metabolic reprogramming. The initial stages of injury exhibit enhanced glycolysis and increased lactate levels, which provide energy; however, the sustained accumulation of lactate may disrupt the dynamic equilibrium of lactate-related epigenetic modifications, thereby affecting immune regulation and the stability of the regenerative microenvironment. Simultaneously, the rise in inflammation-related reactive oxygen species (ROS) may impede the effective activation of myogenic genes by altering the redox state and inhibiting the activity of α-KG-dependent demethylases (García-Giménez et al., 2025). In light of these mechanisms, intervention strategies primarily focus on restoring the dynamic balance between metabolism and epigenetic regulation. These strategies encompass optimizing exercise intensity and restoring rhythm to prevent abnormal metabolite accumulation, regulating inflammation and oxidative stress to maintain enzyme activity, and reconstructing the metabolic environment by enhancing mitochondrial function (Chen et al., 2025). It is essential to recognize that current evidence is largely derived from basic research, and its clinical efficacy necessitates further validation.

The age-related decline in muscle regeneration capacity is closely associated with reduced metabolic function and epigenetic drift. Lower levels of NAD^+^ may inhibit Sirtuin activity, thereby affecting the regulation of deacetylation. Mitochondrial dysfunction can result in decreased production of α-KG, which diminishes the capacity for demethylation. Simultaneously, disruptions in one-carbon metabolism may modify the supply of SAM, thereby influencing the homeostasis of DNA and histone methylation. Collectively, these alterations correlate with decreased MuSC activity and restricted differentiation (Sartorelli and Ciuffoli, 2025). Intervention strategies primarily focus on metabolic remodeling approaches, emphasizing exercise modalities such as resistance and interval training, along with metabolic supplementation strategies that include NAD^+^ precursors and methods to regulate mitochondrial function (Prokopidis et al., 2025; Springer-Sapp et al., 2025). Some epigenetic drugs also show potential value; however, their safety and clinical applicability require further investigation. In the future, stratified interventions should be implemented in conjunction with individual metabolic status.

In metabolic myopathy, abnormal glycolipid metabolism can affect epigenetic regulation through various pathways. Disruptions in glycolysis may interfere with lactate-related signals and modifications. Additionally, disorders in lipid metabolism can hinder the supply of acetyl-CoA, which subsequently impacts histone acetylation levels. Furthermore, abnormalities in one-carbon metabolism may lead to fluctuations in S-adenosylmethionine (SAM), thereby influencing methylation patterns. These alterations are closely linked to the decline in muscle metabolic function and reduced regenerative capacity (Yue et al., 2025; Wang et al., 2026).The intervention strategy advocates for a multidimensional approach that includes enhancing metabolic homeostasis through exercise, optimizing methyl donor levels via nutritional interventions, and regulating energy metabolism and mitochondrial function with pharmacological agents (Kang et al., 2024; Prokopidis et al., 2025). This combined intervention model, which integrates exercise, nutrition, and metabolic regulation, may promote the overall modulation of the metabolic-epigenetic network; however, its underlying mechanisms and clinical effects warrant further investigation. Clinical interventions are summarized in **Table 2**.

**Table 2:** Exercise-based and metabolic interventions targeting the metabolic–epigenetic axis in skeletal muscle.

Intervention	Target metabolic pathway	Epigenetic regulation	Biological effect	Clinical application	References
Endurance exercise	Increased NAD^+^ and α-KG	SIRT1 activation and demethylation	Enhanced mitochondrial function	Metabolic disorders and aging muscle	(Chung et al., 2025; Murach et al., 2025)
Resistance exercise	Increased acetyl-CoA and SAM	Histone acetylation and methylation	Muscle hypertrophy and myonuclear accretion	Sarcopenia and rehabilitation	(Weidenhamer et al., 2025)
Combined training	Multi-pathway activation	Integrated epigenetic modulation	Improved regeneration	Sports injury recovery	(Naeini et al., 2023)
Nutritional modulation	SAM/α-KG availability	DNA and histone methylation	Controlled differentiation	Adjunct therapy	(Murach et al., 2025)

Exercise and metabolic interventions modulate key pathways and epigenetic regulators, influencing muscle regeneration and clinical outcomes.

## Conclusions

Metabolic-epigenetic coupling offers promising opportunities for clinical translation; however, it faces several challenges. First, the causal relationship between metabolic changes and epigenetic regulation remains insufficiently defined. Second, there is a lack of standardized protocols concerning the optimal dosage and sequence of various intervention methods. Furthermore, individual differences, such as age, gender, and basal metabolic status, may significantly affect the efficacy of interventions. Lastly, current clinical evidence is relatively limited, with most studies relying on animal models or *in vitro* experiments. Future research should integrate clinical trials with multi-omics data analysis to develop stratified intervention models based on metabolic-epigenetic characteristics, thereby advancing precision medicine in the context of skeletal muscle regeneration.
